# Effects of Substance Use Disorder on In-Hospital Outcomes of Young Patients Presenting With a Cardiovascular Event: A Nationwide Analysis

**DOI:** 10.7759/cureus.22737

**Published:** 2022-03-01

**Authors:** Ahmed Brgdar, John Gharbin, Ayman Elawad, Jin Yi, Jacob Sanchez, Adey Bishaw, Mohamed E Taha, Edmund Essah Ameyaw, Norman Allen, Mehrotra Prafulla

**Affiliations:** 1 Internal Medicine, Howard University Hospital, Washington, DC, USA; 2 Cardiovascular Medicine, Howard University Hospital, Washington, DC, USA; 3 Department of Mathematics, Howard University College of Medicine, Washington, DC, USA; 4 Cardiovascular Diseases, Providence Hospital, Washington, DC, USA

**Keywords:** alcohol, cannabis, young adults, substance use disorder, cardiac event

## Abstract

Background

Substance use is widely prevalent among young adults and is associated with increased cardiovascular morbidity and mortality such as sudden cardiac arrest, acute coronary syndrome, arrhythmias, and cardiomyopathy. However, they are limited studies analyzing the impact of substance use disorder on in-hospital outcomes among young patients with cardiovascular events.

Methods

All patients aged 18-39 years admitted primarily for major cardiovascular events including acute myocardial infarction (AMI), arrhythmia, cardiac arrest, acute ischemic stroke, and venous thromboembolic events in 2019 were identified in the National Inpatient Sample database. They were then categorized into those with and without concomitant substance use disorder (SUD). The primary outcome was in-hospital mortality. Unadjusted and adjusted analysis was performed on appropriate variables of interest.

Results

Of 57,985 hospitalizations with cardiac events, 12,115 (20%) of young adults had concomitant SUD. SUD was significantly associated with cardiac arrest (OR 3.3; CI 2.4-4.4), atrial fibrillation (OR 1.5; CI 1.3-1.7), AMI (OR 1.3; CI 1.2-1.6), heart failure (OR 2.6; CI 2.4-3.0) (all p<0.05) despite a lower prevalence of traditional cardiovascular risk factors than non-users. Logistic regression showed acute kidney injury (aOR 1.5; CI 1.3-1.8; p<0.001) and inpatient mortality (aOR 1.6; CI 1.2-2.2; p<0.001) were also significantly higher in young patients presenting with cardiac events and concomitant SUD. There was no difference in the length of stay or incidence of gastrointestinal bleed between the two groups.

Conclusion

In young patients presenting with a cardiovascular event, concurrent substance use disorder was associated with increased in-hospital mortality despite significantly lower comorbidities.

## Introduction

Substance abuse has remained a persistent problem in the United States with increasing prevalence. Nearly 101, 263 drug overdose deaths were documented in the 12-month period ending in July 2021, increasing by 76.5% from 57,372 deaths during the same period five years previously [1]. Moreover, substance abuse is disproportionately higher among younger adults in the US. Young adults aged 18-25 years-old report a significantly higher past-month, past-year, and lifetime prevalence of prescription opioids, stimulants, and tranquilizers abuse than older age groups [2] and are the most likely of any age group to die from a prescription drug overdose [3]. Further, a recent analysis of the Veterans Health Administration data showed a 12.8% overall prevalence of substance use disorder (SUD) with a 27.7% prevalence in the group aged 18-34 years old, which was the highest among any age group [4].

Although several studies have reported the cardiovascular outcomes associated with specific substances, many patients with SUD report polysubstance abuse [5,6]. Over 25% of patients with lifetime alcohol use disorder and over 80% of non-alcohol drug-specific SUD (cannabis, cocaine, heroin, hallucinogens, inhalants, prescription opioids, sedatives, tranquilizers, stimulants, or other drugs) report at least one other SUD [6]. As with the prevalence of SUD discussed above, young adults aged 18-29 years old had the highest odds of multiple SUDs [6]. However, there is a paucity of studies assessing the cardiovascular effects of cannabis, stimulants, alcohol, opioid, cocaine, psychoactive agents, hallucinogens, sedatives, hypnotics, and inhalants as a whole. In addition, little is known about the in-hospital outcomes of patients with SUD hospitalized primarily due to a cardiac event [7-9].

The impact of a combination of substances may be clinically more relevant than the effects of an individual substance. For instance, Rumalla et al. concluded that contaminant use of tobacco, cocaine, or amphetamines with cannabis was associated with increased odds of hospitalization for acute ischemic stroke while combining cannabis with opioid or alcohol was associated with lower odds [10]. Similarly, in the recently reported Veterans Affairs Healthcare database study, the risk of premature atherosclerotic cardiovascular diseases was the lowest among cocaine and cannabis users compared to the combinations of amphetamine with cocaine, cannabis, or both [11,12].

Assessing the impact of SUD as a whole becomes even more imperative given that commonly abused substances often have contrasting effects on traditional CVD risk factors. A null association has been reported with cannabis use and central obesity, hyperlipidemia, diabetes, or hypertension [13]. On the other hand, methamphetamine and cocaine abuse had been negatively associated with obesity, hyperlipidemia, and hypertension [9,14,15]. Furthermore, although patients with opioid use disorder tend to have obesity and hyperlipidemia, most remain normotensive [16,17]. In contrast, alcohol increases the risk of cardiovascular diseases by direct action on the cardiovascular system as well as through traditional CVD risk factors, particularly obesity, hypertension, and dyslipidemia [18].

With the objective of bridging some of these gaps, in this study, we have investigated the impact of substance abuse on in-hospital outcomes among patients with cardiovascular events using hospitalization records from the 2019 National Inpatient Sample database (NIS).

## Materials and methods

Data source

We conducted a retrospective cohort study using the NIS database. NIS is the largest inpatient registry in the US with publicly available hospitalization records from 48 participating states and the District of Columbia [19].

Study population

The NIS database was queried using the International Classification of Diseases, Tenth Revision Clinical Modification (ICD-10-CM). Patients aged 18-39 years old admitted primarily for cardiac arrest, atrial fibrillation, and flutter, other cardiac arrhythmias, acute myocardial infarction, pulmonary embolism, heart failure, or acute ischemic stroke in 2019 were identified using the ICD-10-CM codes I46, I48, I49, I21, I26, I50, I63.9, respectively. These constituted cardiac events. From this data, a subset of patients with concomitant alcohol, cocaine, cannabis, hallucinogen, inhalant, opioid, psychoactive agents, stimulants, or sedatives related disorders were identified using the subcategory codes F10, F14, F12, F16, F18, F11, F19, F15, F13, respectively, and combined for substance use disorder. Substance use disorder (SUD) included substance abuse, dependence, use with intoxication, and substance use unspecified according to ICD-10-CM classification. Data were obtained on patient characteristics and hospital-level characteristics. Baseline patient characteristics included age, gender, race, hypertension, diabetes mellitus, obesity, chronic kidney disease, hyperlipidemia, hyperthyroidism. Hospital characteristics including teaching status. Although tobacco use has a strong independent association with adverse cardiovascular outcomes, we excluded patients with tobacco use disorder in line with previous studies [20]. Moreover, recent studies have shown exacerbated cardiovascular outcomes with substances like cannabis and cocaine in the absence of tobacco use compared to patients with concomitant tobacco use disorder [21,22].

Outcomes

The primary outcome of interest was in-hospital mortality. Secondary outcomes of interest were the length of stay, acute kidney injury (AKI), and gastrointestinal hemorrhage.

Statistical analysis

Data were analyzed with Software for Statistics and Data Science (STATA/SE 17.0, Stata Corp, Texas, US). Univariate analyses of cardiac events for the subpopulation were conducted. Multivariate logistic regression was used to adjust for confounders, e.g. age, gender, race, hypertension, diabetes mellitus, obesity, chronic kidney disease, hyperlipidemia, hyperthyroidism, hypothyroidism, nicotine dependence, and Charlson Comorbidity Index (CCI) [23], and hospital teaching status. Continuous variables were expressed as means (95% CI) and used t-tests or regression to compare differences between exposure and non-exposure groups. Similarly, the chi-squared test was used to compare differences between categorical variables. A two-sided p<0.05 was considered significant throughout the analyses.

## Results

A total of 57,985 patients aged 18-39 years old were admitted with cardiac events (including cardiac arrest, atrial fibrillation, and flutter, other cardiac arrhythmias, acute myocardial infarction, pulmonary embolism, heart failure, or acute ischemic stroke) in 2019, out of which 12,115 (20.89%) had SUD. Patients with or without SUD were of similar age (32.7 years vs. 32.5 years), and over half of the patients in both groups were white (52.2% vs. 52.4%) (Table 1). Over 75% of the patients in both groups were treated at teaching hospitals (76.0% vs. 77.0%). Medicaid was the most common insurer for patients with SUD (50.8%), while 48.5% of the patients without SUD were covered by private insurance HMO (Table 1). Among patients with SUD, cannabis was the most widely used substance (26%).

**Table 1:** Baseline demographics and comorbidities of 57,985 young adults aged 18-39 years old admitted with cardiac events with and without substance use disorder (SUD). ǂHMO = Health maintenance organization; *CCI = Charlson Comorbidity Index; #LoS = Length of hospital stay in days; §GI bleed = gastrointestinal hemorrhage

	SUD (n=12,115)	No SUD (n=45,870)	p-value
Mean age, years	32.7 (95% CI:32.6-33.0)	32.5 (95% CI: 32.4-32.7)	0.16
Sex (%)			
Female	28.4%	45.0%	<0.001
Male	71.6%	55.0%	
Race (%)			
White	52.2%	52.4%	<0.001
Black	27.0%	28.2%	
Hispanic	13.7%	12.0%	
Asian/Pacific Islander	2.2%	3.0%	
Native American	2.0%	0.7%	
Other	2.8%	3.7%	
Hospital Characteristics (%)			
Teaching hospital	76.0%	77.0%	0.311
Non-teaching hospital	24.0%	23.0%	
Type of Insurance (%)			
Medicare	6.0%	8.6%	<0.001
Medicaid	50.8%	31.0%	
Private insurance/HMO^ǂ^	23.5%	48.5%	
Self-Pay	19.8%	11.9%	
Type of Substance Use (%)			
Cannabis	26.0%	-	-
Stimulants	23.4%	-	-
Alcohol	20.8%	-	-
Opioid	10.9%	-	-
Cocaine	9.3%	-	-
Psychoactive Agents	9.0%	-	-
Hallucinogens	0.3%	-	-
Sedatives/hypnotics	0.2%	-	-
Inhalant	0.0%	-	-
Comorbidities (%)			
Hypertension	19.7%	23.3%	0.003
Diabetes mellitus	1.7%	2.3%	0.084
Obesity	23.8%	34.9%	<0.001
Chronic kidney disease	5.8%	7.8%	0.002
Hyperlipidemia	14.0%	20.3%	<0.001
Hyperthyroidism	0.9%	1.3%	0.186
Hypothyroidism	2.9%	5.3%	<0.001
Mean CCI^*^	1.45 (95% CI:1.39-1.50)	1.41 (95% CI: 1.38-1.45)	0.281

A greater proportion of patients with SUD were male (71.6% vs. 55.0%). Patients with SUD also had significantly lower prevalence of hypertension (19.7% vs. 23.3%, p=0.003), obesity (23.8% vs. 34.9%, p<0.001), chronic kidney disease (5.8% vs. 7.8%, p=0.002), hyperlipidemia (14.0% vs. 20.3%, p<0.001), and hypothyroidism (2.9% vs. 5.3%, p<0.001) although the mean CCI were similar between patients with or without SUD (1.45 vs. 1.41, p=0.281) (Table 1). The prevalence of diabetes mellitus was numerically lower in the SUD group (1.7% vs. 2.3%, p=0.084).

In the subpopulation univariate analysis, SUD was significantly associated with cardiac arrest (OR = 3.3 (95% CI: 2.4-4.4), p<0.001), atrial fibrillation (OR = 1.5 (95% CI: 1.3-1.7), p<0.001), myocardial infarction (OR = 1.3 (95% CI 1.2-1.6), p<0.001), pulmonary embolism (OR = 1.0 (95% CI: 0.8-1.0), p=0.020), and heart failure (OR = 2.6 (95% CI: 2.4-3.0), p<0.001) but not of other unspecified arrhythmias (OR = 1.2 (95% CI: 1.0-1.5), p=0.074) or acute ischemic stroke (OR = 1.1 (95% CI: 0.9-1.4), p=0.261) (Table 2). 

**Table 2:** Sub-population univariate analysis of cardiac events stratified by substance use disorder

Cardiac Events	Odds Ratio (95% CI)	p-value
Cardiac arrest	3.3 (2.4-4.4)	<0.001
Atrial fibrillation and flutter	1.5 (1.3-1.7)	<0.001
Arrhythmias (atrial fibrillation and atrial flutter excluded)	1.2 (1.0-1.5)	0.074
Myocardial infarction	1.3 (1.2-1.6)	<0.001
Pulmonary embolism	1.0 (0.8-1.0)	0.020
Heart failure	2.6 (2.4-3.0)	<0.001
Acute ischemic stroke	1.1 (0.9-1.4)	0.261

Furthermore, patients presenting with cardiac events with concomitant SUD had a significantly greater risk of in-hospital mortality (OR = 1.5 (95% CI: 1.2-2.0), p<0.001), and acute kidney failure (OR = 1.6 (95% CI: 1.4-1.8), p<0.001). However SUD did not have statistical significant association with gastrointestinal hemorrhage (OR = 1.4 (95% CI: 0.6-2.9), p=0.421) or the length of hospitalization (OR = 0.9 (95% CI: 0.7-1.1), p=0.383) (Table 3). The risk of in-hospital mortality and acute kidney failure remained elevated even after adjusting for potential confounders in the multivariate analysis (Table 3).

**Table 3:** Logistic regression analysis of in-hospital outcomes of 57,985 young adults aged 18-39 years old admitted with cardiac events with and without substance use disorder §GI bleed = gastrointestinal hemorrhage

Outcomes	Univariate		Multivariate	
	Odds Ratio (95% CI)	p-value	Adjusted Odds Ratio (95% CI)	p-value
In-hospital mortality	1.5 (1.2-2.0)	<0.001	1.6 (1.2-2.2)	0.001
Acute kidney failure	1.6 (1.4-1.8)	<0.001	1.5 (1.3-1.8)	<0.001
GI bleed§	1.4 (0.6-2.9)	0.421	1.2 (0.5-2.7)	0.584
Length of stay in days	0.9 (0.7-1.1)	0.383	0.9 (0.7-1.1)	0.452

## Discussion

A growing body of literature suggests that young patients with SUD have exacerbated risk of CVD. A recently published analysis of the nationwide Veterans Affairs Healthcare database showed that young adults with premature atherosclerotic cardiovascular diseases reported significantly higher use of tobacco, alcohol, cocaine, amphetamine, and cannabis, all independently associated with premature atherosclerotic cardiovascular diseases [11]. The same study noted an increased risk of premature atherosclerotic cardiovascular diseases with each additional substance use, and the abuse of ≥4 recreational substances was associated with a nine-fold increase in risk [11]. Our findings expand the scope of these earlier observations by demonstrating that SUD was significantly associated with cardiovascular outcomes, including cardiac arrest, atrial fibrillation, myocardial infarction, pulmonary embolism, and heart failure. Additionally, we observed higher in-hospital mortality and acute kidney failure rates among patients presenting with a cardiovascular event and concomitant SUD.

Our study showed an increased risk of adverse cardiovascular and in-hospital mortality outcomes among SUD patients despite a lower prevalence of traditional CVD risk factors, including hypertension, obesity, and hyperlipidemia. These findings are consistent with previous studies, although earlier studies have focused on single substances, which suggested that commonly abused drugs may produce adverse cardiovascular and mortality outcomes independent of traditional CVD risk factors [13,24,25]. For instance, methamphetamine has been shown to induce cardiomyopathy [26] despite a negative association between methamphetamine abuse and traditional CVD risk factors [14,15]. Also, a significantly higher proportion of cocaine users in the National Cardiovascular Data Registry ACTION Registry-GWTG database had myocardial infarction and cardiogenic shock at presentation despite the lower occurrence of traditional cardiovascular risk factors including obesity, diabetes, dyslipidemia, and hypertension [9]. Cocaine abuse is also known to induce cardiomyopathy, coronary artery spasm, and platelet activation resulting in an increased risk of coronary artery occlusion, myocardial infarction, sudden cardiovascular death, and all-cause mortality, especially in younger patients [27,28].

In contrast, an exacerbated risk of cardiovascular events due to alcohol may be mediated by direct cardiovascular effects of the substances and through traditional CVD risk factors. Alcohol use disorder has been positively associated with several risk factors of CVD, particularly central obesity, hypertension, and hyperlipidemia [16,17,29,30], and has been implicated in the development of atrial fibrillation, non‐ischemic dilated cardiomyopathy, ischemic heart disease, and stroke with a dose-dependent increase in the risk of cardiovascular and all‐cause mortality [31-33].

Opioid abuse may also increase the risk of cardiovascular events through direct action on traditional CVD risk factors, particularly obesity and hyperlipidemia [16,17]. Among patients with opioid overdose-related hospitalizations in the NIS database, 8.6% had at least one cardiovascular event including atrial fibrillation, myocardial infarction, or heart failure episode. There was a significantly higher cost of hospitalization, length of stay, and in-hospital mortality associated with opioid abuse and subsequent cardiovascular events [8]. A recent scoping review that explored the effects of chronic opioid use on CVD outcomes found a positive correlation between chronic opioid use and myocardial infarction [34]. However, other CVD outcomes, such as CAD, arrhythmia, stroke, and heart failure with prescription or non-prescription opioid abuse remain poorly studied [34].

Any potential association between SUD and traditional CVD risk factors or lack thereof must be interpreted along with the strength and limitations of diagnostic criteria used. For example, substance use disorders, specifically alcohol and opioid use are known to increase the constituent risk factors of metabolic syndrome [35,36]. However, several studies indicate that the association between SUD and metabolic syndrome depends on the diagnostic criteria used to define metabolic syndrome with International Diabetes Federation (IDF) criteria, generally associated with positive association, but not National Cholesterol Education Programme Adult Treatment Panel (NCEP ATP-III) criteria [17,36].

Study limitations

Our analysis did not distinguish between abuse of prescription and non-prescription drugs, and we were unable to examine additional confounders such as dose and duration of substance use due to limitations of the NIS database. Moreover, patients' socioeconomic status, which has been associated with the risk of both SUD [37,38] and CVD [39,40] among young adults subpopulations in the US, is another potential confounder not considered in our analysis. While other unmeasured confounders may exist, they are expected to be the same across all groups. Our analysis is limited to in-hospital outcomes of young adults aged 18-39 years and therefore may not generalize to long-term outcomes observed, for example, in older adults or adolescents. Also, the findings of this retrospective observational study need to be confirmed in future randomized control trials. Finally, there is an inherent risk of selection bias in any large database study due to coding errors or missing data, although the National Inpatient Sample auditing process is well established, minimizing data inaccuracy issues.

## Conclusions

In young patients presenting with a cardiovascular event, concurrent substance use disorder was associated with increased in-hospital mortality and acute kidney injury risks despite significantly lower comorbidities. However, no statistically significant difference was observed in length of stay or incidence of gastrointestinal hemorrhage between the two groups. Increasing community collaboration, controlling polysubstance use, and raising awareness of the damaging effects of substance use, especially among young adults is needed.
